# ABodyBuilder3: improved and scalable antibody structure predictions

**DOI:** 10.1093/bioinformatics/btae576

**Published:** 2024-10-03

**Authors:** Henry Kenlay, Frédéric A Dreyer, Daniel Cutting, Daniel Nissley, Charlotte M Deane

**Affiliations:** Exscientia, Oxford OX4 4GE, United Kingdom; Exscientia, Oxford OX4 4GE, United Kingdom; Exscientia, Oxford OX4 4GE, United Kingdom; Exscientia, Oxford OX4 4GE, United Kingdom; Exscientia, Oxford OX4 4GE, United Kingdom; Department of Statistics, University of Oxford, Oxford OX1 3LB, United Kingdom

## Abstract

**Summary:**

In this article, we introduce ABodyBuilder3, an improved and scalable antibody structure prediction model based on ABodyBuilder2. We achieve a new state-of-the-art accuracy in the modelling of CDR loops by leveraging language model embeddings, and show how predicted structures can be further improved through careful relaxation strategies. Finally, we incorporate a predicted Local Distance Difference Test into the model output to allow for a more accurate estimation of uncertainties.

**Availability and implementation:**

The software package is available at https://github.com/Exscientia/ABodyBuilder3 with model weights and data at https://zenodo.org/records/11354577.

## 1 Introduction

Immunoglobulin proteins play a key role in the active immune system, and have emerged as an important class of therapeutics (Lu *et al.* 2020). They are constructed from two heavy and two light chains, separated into distinct domains. The tip of each of the two antibody binding arms is defined as the variable region, and contains six complementarity-determining regions (CDRs) across the heavy and light chains which make up most of the antigen-binding site. As part of an immune response, B cells undergo clonal expansion, which, coupled with somatic hypermutations and recombinations, leads to an accumulation of mutations in the DNA encoding the CDR loops. The remaining domains compose the constant region and are primarily involved in effector function.

Understanding the 3D structure of antibodies is critical to assessing their properties (Chungyoun and Gray 2023) and developability (Raybould *et al.* 2019, 2024). The framework regions connecting the CDR loops are relatively conserved and thus easily predicted from sequence similarity. Similarly, five of the CDR loops tend to cluster along canonical forms (Adolf-Bryfogle *et al.* 2014, Wong *et al.* 2019) and are thus relatively straightforward to model. The third loop of the heavy chain (CDRH3), for which the coding sequence is created during the recombination of the V, D, and J gene segments (Roth 2014), is however more challenging due to its much larger sequence and length diversity. As the CDRH3 loop often drives antigen recognition, e.g. (Narciso *et al.* 2011, Tsuchiya and Mizuguchi 2016), improving the accuracy with which its structure can be predicted from sequence is a key component to advancing rational antibody design.

Experimental protein structure determination remains a costly and slow process (Slabinski *et al.* 2007), such that only a small fraction of known antibody sequences have experimentally resolved 3D structural information (Dunbar *et al.* 2014, Schneider *et al.* 2022). One approach to circumvent these experimental limitations is through structure prediction methods, which have had immense success in reaching experimental accuracy on general protein structures (Baek *et al.* 2021, Jumper *et al.* 2021, Lin *et al.* 2023).

Structure models are also a necessary element to accurately predict biophysical properties of proteins and advance the field of rational therapeutic design. Several dedicated tools have emerged to model specifically the variable region of antibodies. Among them are IgFold (Ruffolo *et al.* 2023), which is based on a language model, DeepAb (Ruffolo *et al.* 2022), which uses an attention mechanism, ABlooper (Abanades *et al.* 2022), which predicts backbone atom positions using a graph neural network, ABodyBuilder (Leem *et al.* 2016), a homology modelling pipeline, and ABodyBuilder2 (Abanades *et al.* 2023), which uses a modified version of the AlphaFold-Multimer architecture (Evans *et al.* 2022). More recently, xTrimoPGLM-Ab (Chen *et al.* 2023) has shown promising results on antibody structures by combining a General Language Model framework (Zeng *et al.* 2023) with a modified AlphaFold2 architecture.

In this article, we introduce ABodyBuilder3, an antibody structure prediction model based on ABodyBuilder2 (Abanades *et al.* 2023). We detail key changes to the implementation, data curation, sequence representation and structure refinement that improve the scalability and accuracy of the model. Additionally, we introduce an uncertainty estimation based on the predicted local-distance difference test (pLDDT), which outperforms the previous ensemble-based estimate. Together, these enhancements provide a substantial improvement in the quality of antibody structure predictions and open the possibility of a scalable and precise assessment of large numbers of therapeutic candidates.

## 2 Model overview

**Figure 1.**
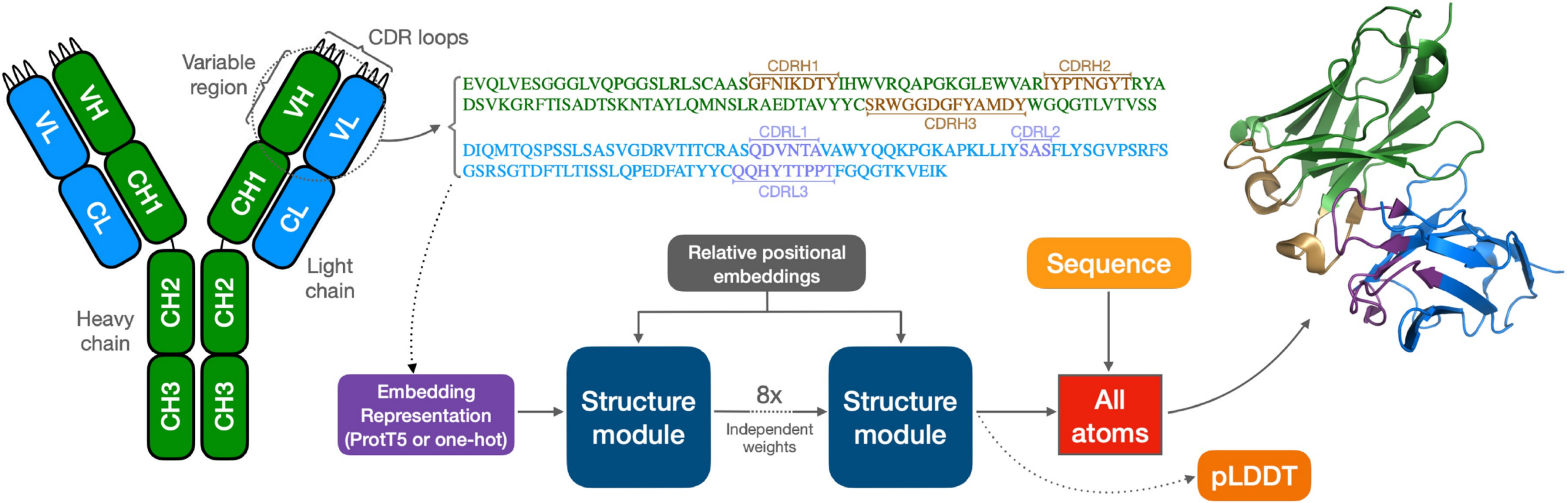
Left: Overview of an antibody structure, with the variable region and CDR loops shown. Right: An embedding representation of the variable region is created by concatenating the heavy and light chain variable region one-hot encoding or ProtT5 embedding. This is given as input to eight sequential update blocks with independent weights. The output of the final update block is used to predict the final backbone atomic coordinates and uncertainties. Full sequence information is then used to predict chi-angles and reconstruct all side-chain atoms using idealized coordinates.

An overview of the ABodyBuilder3 architecture, which is comparable to the ABodyBuilder2 model, is shown in Fig. 1. The input node features consist of an embedding representation of the variable region sequence, where the residue one-hot encoding or the ProtT5 embedding of the heavy and light chain variable sequences are concatenated. Relative positional encodings are used as edge features. This graph is provided as input to a sequence of eight structure modules that update the node features and residue coordinates through an invariant point attention layer and a backbone update layer, respectively, starting from residues set at the origin (Jumper *et al.* 2021). The final layer of the structure module is used in conjunction with the input variable region sequence to predict all atom coordinates by generating torsion angles from the node features.

To train the model, a Frame Aligned Point Error (FAPE) loss is used along with structural violation and backbone torsion angle losses. As in ABodyBuilder2, the FAPE loss is clamped at 30 Å between CDR and framework residues and at 10 Å otherwise, with the final FAPE loss term computed as the sum of the average backbone FAPE loss after each backbone update and the full atom FAPE loss from the final prediction. Training is performed in two stages using a batch size of 64, with the first stage only using the FAPE and torsion angle losses. We use the RAdam optimizer (Liu *et al.* 2021) with warm restart every 50 epochs using a cosine annealing scheduler. The second stage incorporates the structural violation term to the loss and uses a fixed learning rate of $10^{-4}$. For both stages, training is stopped after the validation loss stops improving for 100 epochs, and the best model checkpoint is used.

## 3 Improved structure modelling and evaluation

Rapid prototyping is paramount to generating insights and improving the design of machine learning models. We develop an efficient and scalable implementation of the ABodyBuilder2 architecture which makes use of vectorization to improve hardware utilization, leveraging optimisations from the OpenFold project (Ahdritz *et al.* 2024). This is in contrast to the implementation of ABodyBuilder2, which generates minibatch gradients by computing a gradient for each minibatch sample sequentially before averaging (i.e. accumulated gradients). ABodyBuilder2 also uses double precision which is not well optimized on modern GPU hardware compared to lower precision data-types. We find the model can be trained robustly using bfloat16 precision for weights and use mixed precision for training, providing faster computational throughput and an efficient memory footprint. Our implementation is more than three times faster, and can be scaled easily across multiple GPUs using a distributed data parallel strategy.

We use the Structural Antibody Database (SAbDab) (Dunbar *et al.* 2014), a dataset of experimentally resolved antibody structures, to train our models on all available data up to January 2024. We perform an initial filtering to remove nanobodies, structures with resolution above 3.5 Å, and outliers >3.5 standard deviations from the mean for any of the six summary statistics given by ABangle (Dunbar *et al.* 2013). Furthermore, we filter out ultra-long CDRH3 loops, which predominantly come from bovine antibodies (de Los Rios *et al.* 2015) by removing any sequence with a CDRH3 of over 30 residues. We label residues using IMGT numbering generated via ANARCI (Dunbar and Deane 2016). In an attempt to remove potential structural outliers, we also remove antibodies from species which occur >15 times in SAbDab.

For both the first and second stage of training we select weights based on the lowest validation loss. We use a validation set of 150 structures and a test set of 100 structures, which are significantly larger than those used in ABodyBuilder2 and lead to a more robust assessment of model capabilities. We retain the original ABodyBuilder2 test set of 34 structures as a subset of our test set to allow for direct comparisons. As a primary interest is the modelling of antibodies with high humanness in the context of therapeutic antibody development, we constrain the validation and additional test structures to be annotated as human. We require a resolution below 2.5 Å and a CDRH3 length of >22 for the selection of our validation structures. Furthermore, we remove any structures from the training data that share an identical sequence in any of the CDR regions with any of the validation or test sets.

We consider two physics-based refinement strategies, OpenMM (Eastman *et al.* 2017) and YASARA (Krieger and Vriend 2015), to fix stereochemical errors and provide realistic structures. We find that minimization in the YASARA2 forcefield (Krieger *et al.* 2009) in explicit water leads to improved accuracy of all regions, particularly in the framework. Further details and comparisons between the minimization methods are given in Supplementary Information S1.

In Table 1, the first three rows show a comparison of the original ABodyBuilder2 model with our baseline model obtained with our improved implementation and dataset curation. We give the root mean squared deviation (RMSD) for each region of the variable domain, and provide results with both refinement strategies. Here the RMSD is computed by aligning the heavy and light chains separately to the crystal structure and averaging the RMSD of backbone atoms over the residues of each CDR and framework region. Note here that ABodyBuilder2 predictions are obtained by taking the closest structure to the mean of an ensemble of four models. This ensemble of models is selected from ten distinct trainings of which six models are then discarded. By comparison, our baseline consists of a single model without any need for model selection and ensemble prediction. The full RMSD distributions are given in Supplementary Information S2.

**Table 1.** Modelling accuracy as measured by mean RMSD in Angstroms, given for each CDR loop and framework region.^a^

	CDRH1	CDRH2	CDRH3	Fw-H	CDRL1	CDRL2	CDRL3	Fw-L
ABodyBuilder2	0.84	0.73	2.54	0.56	0.55	0.36	0.88	0.53
Baseline (OpenMM)	0.92	0.75	2.53	0.60	0.67	0.35	0.96	0.58
Baseline (Yasara)	0.90	0.74	2.49	0.59	0.58	0.37	0.92	0.57
ABodyBuilder3	0.87	0.70	2.42	0.58	0.61	0.39	0.93	0.58
ABodyBuilder3-LM	0.87	0.75	2.40	0.57	0.59	0.37	0.89	0.58

a Baseline models refer to a single model with our optimized implementation of the ABodyBuilder2 architecture trained on our updated curated dataset whereas ABodyBuilder2 refers to the original version which uses an ensemble of models and the prediction closest to the mean.

## 4 Language model representation

Inspired by the success of language model embeddings being used to model protein structure, e.g. (Lin *et al.* 2023, Ruffolo *et al.* 2023), we experiment with replacing the one-hot-encoding used as the residue representation in ABodyBuilder2 with a language model embedding. We use the ProtT5 model (Elnaggar *et al.* 2021), an encoder-decoder text-to-text transformer model (Raffel *et al.* 2020) pretrained on billions of protein sequences, to generate a residue level embedding of each antibody. As this language model was trained on single chains, we embed the heavy and light chain separately by passing them through the ProtT5 model, and concatenate their residue representations to obtain a per-residue embedding of the full variable region. We also explored antibody-specific models such as the paired IgT5 and IgBert models (Kenlay *et al.* 2024), but ultimately found that general protein language models achieved higher performance. This might be because antibody language models introduce potential dataset contamination and overfitting during the language model pre-training. To train our language model-based structure prediction model, all parameters are kept identical to ABodyBuilder2, except for a lower initial learning rate of $5\cdot 10^{-4}$, and a reduction of the minimum learning rate to 0 in the scheduler, which we found to improve stability on learning rate resets.

In Table 1, we show the performance of our ABodyBuilder3 model, comparing the one-hot encoding with the ProtT5 embedding representation which we denote as ABodyBuilder3-LM. One can observe a small reduction in RMSD using the language model representation, notably in the modelling of the CDRH3 and CDRL3 loops, though this improvement is not statistically significant.

## 5 Uncertainty estimation

The ABodyBuilder2 model uses an ensemble of four models to provide a confidence score from the diversity between predictions. This approach has an increased computational burden, as multiple models are required both at training and inference time. We instead estimate the intrinsic model accuracy by predicting the per-residue lDDT-Cα scores (Mariani *et al.* 2013), as implemented in the AlphaFold2 model (Jumper *et al.* 2021). This introduces a very small increase in the number of parameters, but circumvents the need for an ensemble of models. The pLDDT is obtained from the final single representation of the structure module. A multilayer perceptron with softmax activation predicts a projection of the local confidence into 50 bins. Training is achieved by discretising the predicted structure with per-residue lDDT-Cα against the ground truth structure and computing the cross-entropy loss, which is added to the original ABodyBuilder2 loss with a weight of 0.01. A pLDDT score for the full variable domain, or for specific regions, is obtained as an average of the corresponding per-residue pLDDT scores.

**Figure 2.**
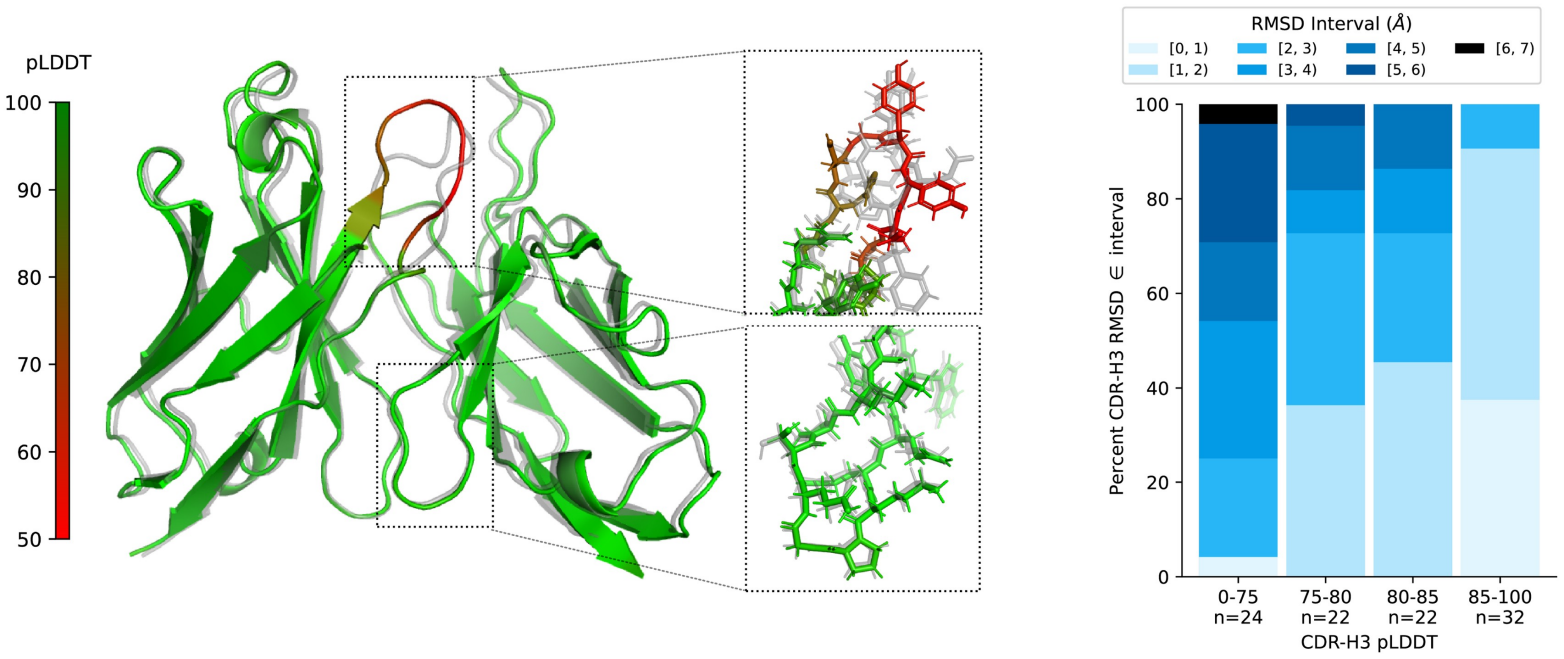
Left: Structure predicted by ABodyBuilder3, with colouring indicating the pLDDT uncertainty estimate. The ground truth (7T0J) is shown in grey. Right: Distribution of CDRH3 RMSD across different bins of the CDRH3 pLDDT score.

In Table 2, we give the Pearson correlation between the pLDDT score and the RMSD, averaged over each region of the variable domain. The ABodyBuilder2 model, with an uncertainty score obtained from the ensemble model, has lower correlation with RMSD than our pLDDT score. It is interesting to note here that the ABodyBuilder3-LM model, which uses ProtT5 embeddings as input, achieves a higher correlation than the one-hot encoding representation model, notably in the modelling of the CDRH3 uncertainty. We note however that when considering the Spearman correlation, shown in Supplementary Information S3, the difference between models is less marked. We provide a guideline for thresholding pLDDT for modelling the CDRH3 region in Fig. 2 (right), applied here on the full test set. Incorporating a threshold of a pLDDT above 85, we retain approximately 32% of structures, with over 80% of those retained having a CDR-H3 RMSD below 2 Å.

**Table 2.** Pearson correlation between average uncertainty prediction for a region and the corresponding mean RMSD.^a^

	CDRH1	CDRH2	CDRH3	Fw-H	CDRL1	CDRL2	CDRL3	Fw-L
ABodyBuilder2	0.41	0.38	0.57	0.50	0.47	0.48	0.72	0.40
ABodyBuilder3	0.58	0.26	0.61	0.48	0.60	0.20	0.68	0.67
ABodyBuilder3-LM	0.69	0.36	0.73	0.39	0.72	0.52	0.68	0.58

a Uncertainties for ABodyBuilder2 are derived from an ensemble of four models, while all ABodyBuilder3 uncertainties are directly predicted by a pLDDT head.

## 6 Conclusions

In this article, we present ABodyBuilder3, a state-of-the-art antibody structure prediction model based on ABodyBuilder2. We incorporated several improvements to the implementation, notably enhancing hardware acceleration through vectorization, which significantly improve the scalability of our model. We also made changes to the data processing and structure refinement that lead to more accurate predictions.

In addition, we show how leveraging a language model representation of the antibody sequence can improve the modelling of CDRH3. Though the improvement in RMSD is marginal, our model achieves more robust training. Finally, we demonstrate how the use of pLDDT head, combined with protein language model embeddings, can be used as a substitute for an ensemble of models approach, which require substantially more training and inference compute.

It would be interesting to explore the use of self-distillation, which has shown to improve accuracy in general protein structure prediction models (Jumper *et al.* 2021), by pre-training our model on a large dataset of synthetic structures predicted from the paired Observed Antibody Space (Kovaltsuk *et al.* 2018, Greenshields-Watson *et al.* 2024). To even further improve the accuracy of the predictions and of the uncertainty estimates, one could also consider combining the pLDDT approach introduced in this article with an ensemble of models, though this would be at the cost of increased training and inference compute.

## Supplementary Material

btae576_Supplementary_Data

## Data Availability

Code is available at https://github.com/Exscientia/abodybuilder3. Data and model weights are available at https://zenodo.org/records/11354577.

## References

[btae576-B1] Abanades B , Georges G, Bujotzek A et al ABlooper: fast accurate antibody CDR loop structure prediction with accuracy estimation. Bioinformatics 2022;38:1877–80. 35099535 10.1093/bioinformatics/btac016 PMC8963302

[btae576-B2] Abanades B , Wong WK, Boyles F et al Immunebuilder: deep-learning models for predicting the structures of immune proteins. Commun Biol 2023;6:575. 37248282 10.1038/s42003-023-04927-7 PMC10227038

[btae576-B3] Adolf-Bryfogle J , Xu Q, North B Jr et al PyIgClassify: a database of antibody CDR structural classifications. Nucleic Acids Res 2014;43:D432–8. 25392411 10.1093/nar/gku1106 PMC4383924

[btae576-B4] Ahdritz G , Bouatta N, Kadyan S et al Openfold: retraining alphafold2 yields new insights into its learning mechanisms and capacity for generalization. Nat Methods 2024;21:1514–24. 38744917 10.1038/s41592-024-02272-z PMC11645889

[btae576-B5] Baek M , DiMaio F, Anishchenko I et al Accurate prediction of protein structures and interactions using a three-track neural network. Science 2021;373:871–6. 34282049 10.1126/science.abj8754 PMC7612213

[btae576-B6] Chen B , Cheng X, Ao Geng Y et al xTrimoPGLM: unified 100b-scale pre-trained transformer for deciphering the language of protein. bioRxiv, 10.48550/arXiv.2401.06199, 2023, preprint: not peer reviewed.

[btae576-B7] Chungyoun M , Gray JJ. Ai models for protein design are driving antibody engineering. Curr Opin Biomed Eng 2023;28:100473. 37484815 10.1016/j.cobme.2023.100473 PMC10361400

[btae576-B8] de Los Rios M , Criscitiello MF, Smider VV. Structural and genetic diversity in antibody repertoires from diverse species. Curr Opin Struct Biol 2015;33:27–41. 26188469 10.1016/j.sbi.2015.06.002 PMC7039331

[btae576-B9] Dunbar J , Deane CM. Anarci: antigen receptor numbering and receptor classification. Bioinformatics 2016;32:298–300. 26424857 10.1093/bioinformatics/btv552 PMC4708101

[btae576-B10] Dunbar J , Fuchs A, Shi J et al Abangle: characterising the VH–VL orientation in antibodies. Protein Eng Des Sel 2013;26:611–20. 23708320 10.1093/protein/gzt020

[btae576-B12] Dunbar J , Krawczyk K, Leem J et al SAbDab: the structural antibody database. Nucleic Acids Res 2014;42:D1140–6. 24214988 10.1093/nar/gkt1043 PMC3965125

[btae576-B13] Eastman P , Swails J, Chodera JD et al Openmm 7: rapid development of high performance algorithms for molecular dynamics. PLoS Comput Biol 2017;13:e1005659. 28746339 10.1371/journal.pcbi.1005659 PMC5549999

[btae576-B14] Elnaggar A , Heinzinger M, Dallago C et al Prottrans: toward understanding the language of life through self-supervised learning. IEEE Trans Pattern Anal Mach Intell 2021;44:7112–27. 10.1109/TPAMI.2021.3095381 34232869

[btae576-B15] Evans R , Michael Neill A, Pritzel N et al Protein complex prediction with AlphaFold-multimer. bioRxiv, 10.1101/2021.10.04.463034, 2022, preprint: not peer reviewed.

[btae576-B16] Greenshields-Watson A , Abanades B, Deane CM. Investigating the ability of deep learning-based structure prediction to extrapolate and/or enrich the set of antibody CDR canonical forms. Front Immunol 2024;15. 10.3389/fimmu.2024.1352703. PMC10933040 38482007

[btae576-B17] Jumper J , Evans R, Pritzel A et al Highly accurate protein structure prediction with alphafold. Nature 2021;596:583–9. 34265844 10.1038/s41586-021-03819-2 PMC8371605

[btae576-B18] Kenlay H , Dreyer FA, Kovaltsuk A et al Large scale paired antibody language models. bioRxiv, https://arxiv.org/abs/2403.17889, 2024, preprint: not peer reviewed. 10.1371/journal.pcbi.1012646 PMC11654935 39642174

[btae576-B19] Kovaltsuk A , Leem J, Kelm S et al Observed antibody space: a resource for data mining next-generation sequencing of antibody repertoires. J Immunol 2018;201:2502–9. 30217829 10.4049/jimmunol.1800708

[btae576-B20] Krieger E , Vriend G. New ways to boost molecular dynamics simulations. J Comput Chem 2015;36:996–1007. 25824339 10.1002/jcc.23899 PMC6680170

[btae576-B21] Krieger E , Joo K, Lee J et al Improving physical realism, stereochemistry, and side-chain accuracy in homology modeling: four approaches that performed well in casp8. Proteins Struct Funct Bioinf 2009;77:114–22. 10.1002/prot.22570 PMC2922016 19768677

[btae576-B22] Leem J , Dunbar J, Georges G et al ABodyBuilder: automated antibody structure prediction with data–driven accuracy estimation. MAbs 2016;8:1259–68. 27392298 10.1080/19420862.2016.1205773 PMC5058620

[btae576-B23] Lin Z , Akin H, Rao R et al Evolutionary-scale prediction of atomic-level protein structure with a language model. Science 2023;379:1123–30. 36927031 10.1126/science.ade2574

[btae576-B24] Liu L , Jiang H, He P et al On the variance of the adaptive learning rate and beyond. 2021. https://arxiv.org/abs/1908.03265.

[btae576-B25] Lu R-M , Hwang Y-C, Liu I-J et al Development of therapeutic antibodies for the treatment of diseases. J Biomed Sci 2020;27:1. 31894001 10.1186/s12929-019-0592-z PMC6939334

[btae576-B26] Mariani V , Biasini M, Barbato A et al lDDT: a local superposition-free score for comparing protein structures and models using distance difference tests. Bioinformatics 2013;29:2722–8. 23986568 10.1093/bioinformatics/btt473 PMC3799472

[btae576-B27] Narciso JE , Uy I, Cabang A et al Analysis of the antibody structure based on high-resolution crystallographic studies. N Biotechnol 2011;28:435–47. 21477671 10.1016/j.nbt.2011.03.012

[btae576-B28] Raffel C , Shazeer N, Roberts A et al Exploring the limits of transfer learning with a unified text-to-text transformer. J Mach Learn Res 2020;21:1−67.34305477

[btae576-B29] Raybould MIJ , Marks C, Krawczyk K et al Five computational developability guidelines for therapeutic antibody profiling. Proc Natl Acad Sci USA 2019;116:4025–30. 30765520 10.1073/pnas.1810576116 PMC6410772

[btae576-B30] Raybould MIJ , Turnbull OM, Suter A et al Contextualising the developability risk of antibodies with lambda light chains using enhanced therapeutic antibody profiling. Commun Biol 2024;7:62. 38191620 10.1038/s42003-023-05744-8 PMC10774428

[btae576-B31] Roth DB. V(D)J recombination: mechanism, errors, and fidelity. Microbiol Spectr 2014;2. https://journals.asm.org/doi/10.1128/microbiolspec.mdna3-0041-2014. 10.1128/microbiolspec.MDNA3-0041-2014 PMC5089068 26104458

[btae576-B32] Ruffolo JA , Sulam J, Gray JJ. Antibody structure prediction using interpretable deep learning. Patterns (N Y) 2022;3:100406. 35199061 10.1016/j.patter.2021.100406 PMC8848015

[btae576-B33] Ruffolo JA , Chu L-S, Pooja Mahajan S et al Fast, accurate antibody structure prediction from deep learning on massive set of natural antibodies. Nat Commun 2023;14:2389. 37185622 10.1038/s41467-023-38063-x PMC10129313

[btae576-B34] Schneider C , Raybould MIJ, Deane CM. SAbDab in the age of biotherapeutics: updates including SAbDab-nano, the nanobody structure tracker. Nucleic Acids Res 2022;50:D1368–72. 34986602 10.1093/nar/gkab1050 PMC8728266

[btae576-B35] Slabinski L , Jaroszewski L, Rodrigues APC et al The challenge of protein structure determination—lessons from structural genomics. Protein Sci 2007;16:2472–82. 17962404 10.1110/ps.073037907 PMC2211687

[btae576-B36] Tsuchiya Y , Mizuguchi K. The diversity of h3 loops determines the antigen-binding tendencies of antibody cdr loops. Protein Sci 2016;25:815–25. 26749247 10.1002/pro.2874 PMC4941225

[btae576-B37] Wong WK , Leem J, Deane CM. Comparative analysis of the cdr loops of antigen receptors. Front Immunol 2019;10:2454–3224. 31681328 10.3389/fimmu.2019.02454 PMC6803477

[btae576-B38] Zeng A , Liu X, Du Z et al GLM-130B: an open bilingual pre-trained model. 2023. https://arxiv.org/abs/2210.02414.

