# Supplementary material for: ABodyBuilder3: improved and scalable antibody structure predictions
Source: Bioinformatics. 2024 Oct 3;40(10):btae576. doi: 10.1093/bioinformatics/btae576 (PMC11474105; doi:10.1093/bioinformatics/btae576)
Supplement: btae576_Supplementary_Data [file btae576_supplementary_data.pdf]

ABodyBuilder3: Supplementary Information

1 Structure refinement

Most approaches to protein or antibody structure prediction, including AlphaFold2 (Jumper et al., 2021), AlphaFold2-Multimer (Evans et al., 2022), and ABodyBuilder2 (Abanades et al., 2022), that predominantly rely on deep learning also use a final physics-based refinement step to fix stereochemical errors and provide realistic structures. In most cases, this refinement takes the form of an in-vacuo minimization that may neglect important aspects of the real system in favor of expediency. To test the influence of different types of minimization on the quality of ABodyBuilder3 output structures, we compare an OpenMM refinement with a refinement using YASARA2, shown in Figure 1. These results confirm that while minimization in vacuo is sufficient to improve many structures, a minimization in the YASARA2 forcefield (Krieger et al., 2009; Krieger and Vriend, 2015) in explicit water allows for further improvements across all the regions, particularly in the framework, whilst improving model quality according to the z-score produced by YASARA2.

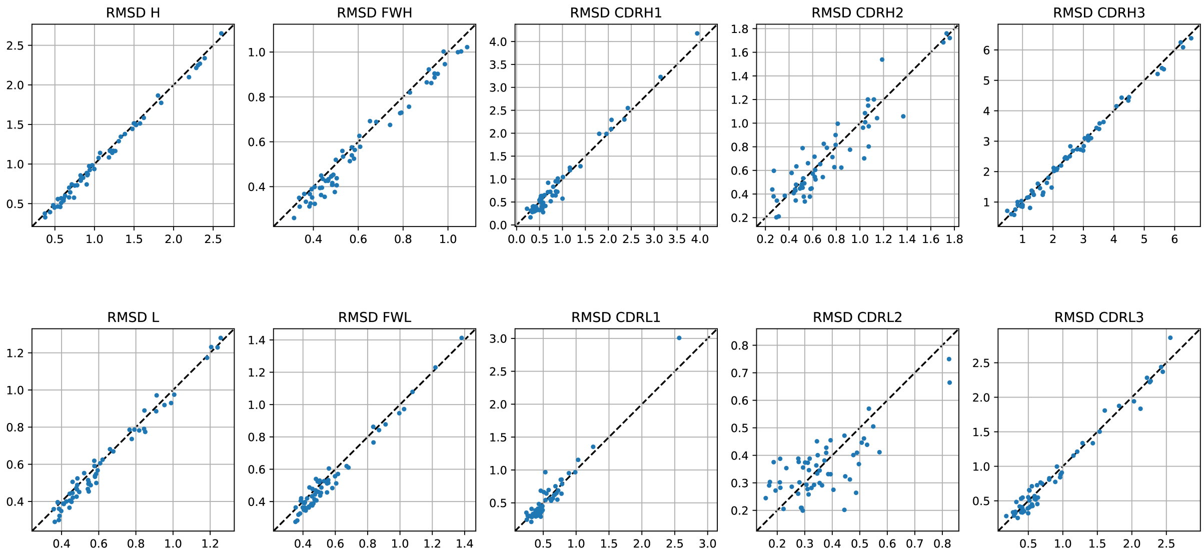

Figure 1: YASARA2 refinement (x-axis) compared to OpenMM refinement (y-axis).

2 RMSD distributions

We show the distribution of the test set RMSD values for ABodyBuilder2, our openMM baseline, ABodyBuilder3 and ABodyBuilder3-LM in Fig. 2.

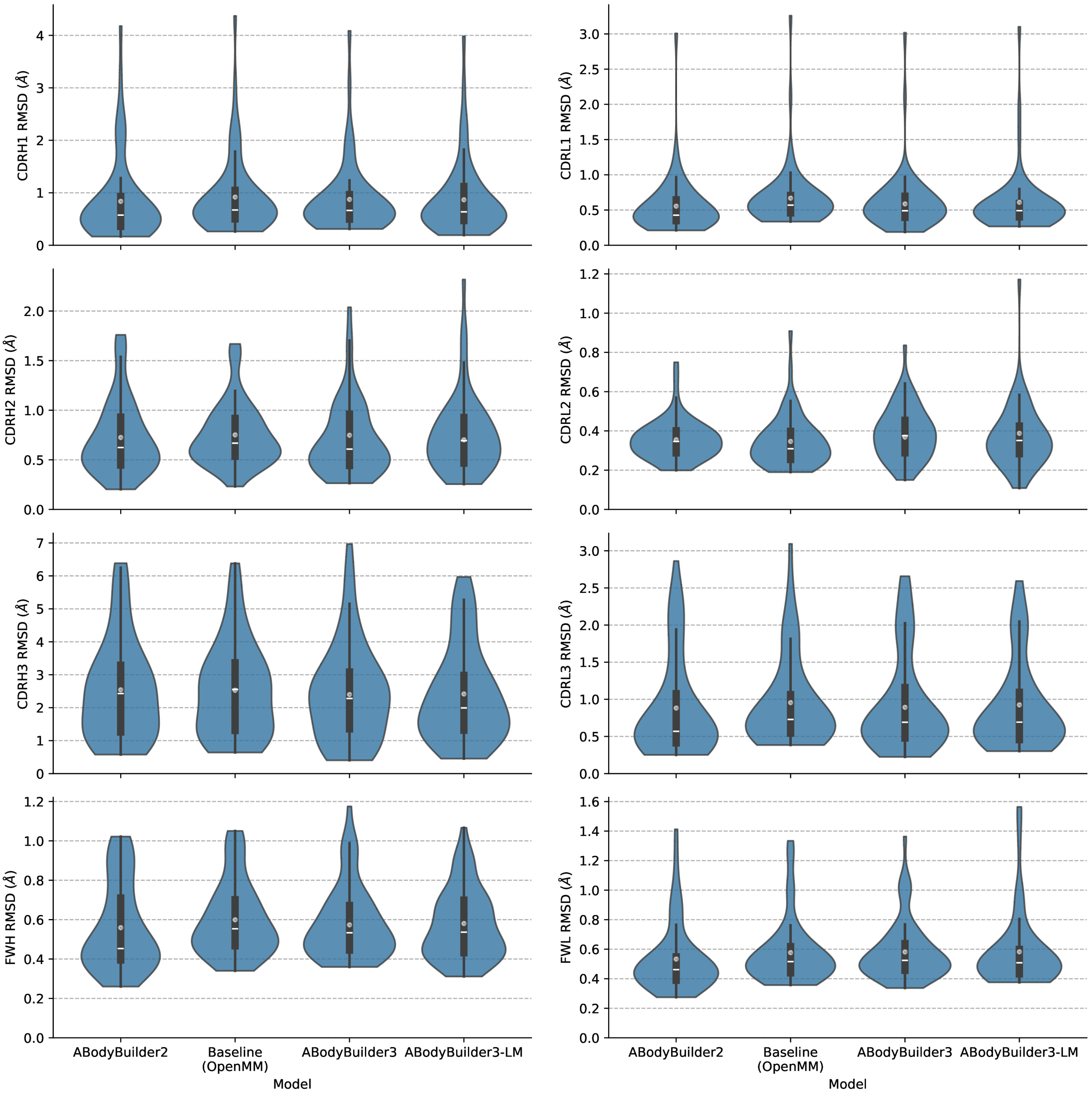

Figure 2: Distribution of the RMSD between predicted and true complexes in the test set, split according to region and model. The interior of each violin plot contains a box plot where the median of the distribution is shown as a horizontal white line and the mean value is show as a gray circle.

3 Spearman correlation of uncertainties

We show the Spearman correlation between uncertainty predictions and RMSD in Table 1.

	CDRH1	CDRH2	CDRH3	Fw-H	CDRL1	CDRL2	CDRL3	Fw-L
ABodyBuilder2	0.45	0.30	0.75	0.50	0.56	0.21	0.73	0.42
ABodyBuilder3	0.48	0.23	0.63	0.50	0.37	0.01	0.59	0.59
ABodyBuilder3-LM	0.48	0.40	0.73	0.27	0.43	0.20	0.60	0.53

Table 1: Spearman correlation between average uncertainty prediction for a region and the corresponding mean RMSD.

References

Brennan Abanades, Wing Ki Wong, Fergus Boyles, Guy Georges, Alexander Bujotzek, and Charlotte M. Deane. Immunebuilder: Deep-learning models for predicting the structures of immune proteins. *bioRxiv*, 2022.

Richard Evans, Michael O'Neill, Alexander Pritzel, Natasha Antropova, Andrew Senior, Tim Green, Augustin Žídek, Russ Bates, Sam Blackwell, Jason Yim, Olaf Ronneberger, Sebastian Bodenstein, Michal Zielinski, Alex Bridgland, Anna Potapenko, Andrew Cowie, Kathryn Tunyasuvunakool, Rishub Jain, Ellen Clancy, Pushmeet Kohli, John Jumper, and Demis Hassabis. Protein complex prediction with alphafold-multimer. *bioRxiv*, 2022.

John Jumper, Richard Evans, Alexander Pritzel, Tim Green, Michael Figurnov, Olaf Ronneberger, Kathryn Tunyasuvunakool, Russ Bates, Augustin Žídek, Anna Potapenko, Alex Bridgland, Clemens Meyer, Simon A. A. Kohl, Andrew J. Ballard, Andrew Cowie, Bernardino Romera-Paredes, Stanislav Nikolov, Rishub Jain, Jonas Adler, Trevor Back, Stig Petersen, David Reiman, Ellen Clancy, Michal Zielinski, Martin Steinegger, Michalina Pacholska, Tamas Berghammer, Sebastian Bodenstein, David Silver, Oriol Vinyals, Andrew W. Senior, Koray Kavukcuoglu, Pushmeet Kohli, and Demis Hassabis. Highly accurate protein structure prediction with alphafold. *Nature*, 596(7873):583–589, 2021.

Elmar Krieger and Gert Vriend. New ways to boost molecular dynamics simulations. *Journal of Computational Chemistry*, 36(13):996–1007, 2015.

Elmar Krieger, Keehyoung Joo, Jinwoo Lee, Jooyoung Lee, Srivatsan Raman, James Thompson, Mike Tyka, David Baker, and Kevin Karplus. Improving physical realism, stereochemistry, and side-chain accuracy in homology modeling: four approaches that performed well in casp8. *Proteins: Structure, Function, and Bioinformatics*, 77(S9):114–122, 2009.
